# ANO1: More Than Just Calcium-Activated Chloride Channel in Cancer

**DOI:** 10.3389/fonc.2022.922838

**Published:** 2022-06-06

**Authors:** Saisai Guo, Linna Zhang, Na Li

**Affiliations:** Department of Oncology, The Second Affiliated Hospital of Dalian Medical University, Dalian, China

**Keywords:** ANO1, cancer, protein network, signal pathway, tumor microenvironment, inhibitor, miRNA

## Abstract

ANO1, a calcium-activated chloride channel (CACC), is also known as transmembrane protein 16A (TMEM16A). It plays a vital role in the occurrence, development, metastasis, proliferation, and apoptosis of various malignant tumors. This article reviews the mechanism of ANO1 involved in the replication, proliferation, invasion and apoptosis of various malignant tumors. Various molecules and Stimuli control the expression of ANO1, and the regulatory mechanism of ANO1 is different in tumor cells. To explore the mechanism of ANO1 overexpression and activation of tumor cells by studying the different effects of ANO1. Current studies have shown that ANO1 expression is controlled by 11q13 gene amplification and may also exert cell-specific effects through its interconnected protein network, phosphorylation of different kinases, and signaling pathways. At the same time, ANO1 also resists tumor apoptosis and promotes tumor immune escape. ANO1 can be used as a promising biomarker for detecting certain malignant tumors. Further studies on the channels and the mechanism of protein activity of ANO1 are needed. Finally, the latest inhibitors of ANO1 are summarized, which provides the research direction for the tumor-promoting mechanism of ANO1.

## Introduction

ANO1, as a calcium-activated chloride channel (CaCC), also known as transmembrane protein 16A (TMEM16A), is a voltage-sensitive calcium-activated chloride channel with ten transmembrane fragments with amino and carboxyl-terminal structure (1). It is widely expressed in many cells, including epithelial cells, airways, smooth muscle cells, vascular endothelial cells, and myocardium. ANO1 also modulates several physiological functions, such as fluid and electrolyte secretion, intestinal motility, cardiac and neuronal excitability, vascular smooth muscle contraction, and heat pain (2, 3). Abnormal circulating ANO1 is associated with susceptibility and pathogenesis of several human diseases and pathological entities, including cystic fibrosis, various cancers, hypertension, and gastrointestinal motility disorders (4–6).

ANO1 also has a crucial role in tumor occurrence, development, metastasis, proliferation, anti-apoptosis, and epigenetic regulation is often overexpressed in tumor tissues and is an experimental target for antitumor therapies. However, the biological functions of malignant tumors remain a controversial issue. The present review aims to explore the role of ANO1 in cancer pathogenesis and therapeutics and to elucidate the mechanisms underlying the relation between ANO1 and malignancy.

## Ano1 as A Potential Biomarker In Cancer

**Figure 1:**
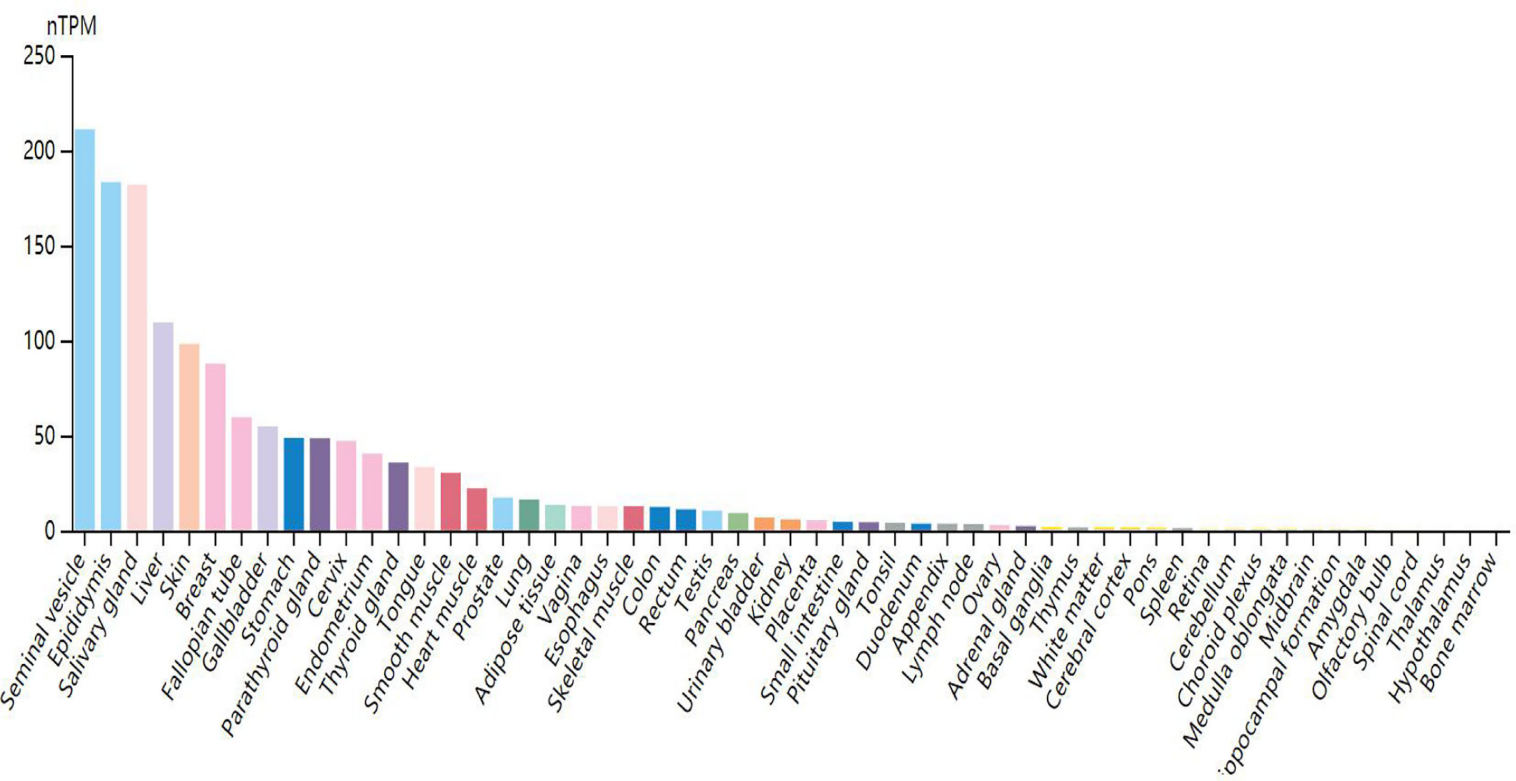
Expression level of ANO1 in tissues.

**Figure 2:**
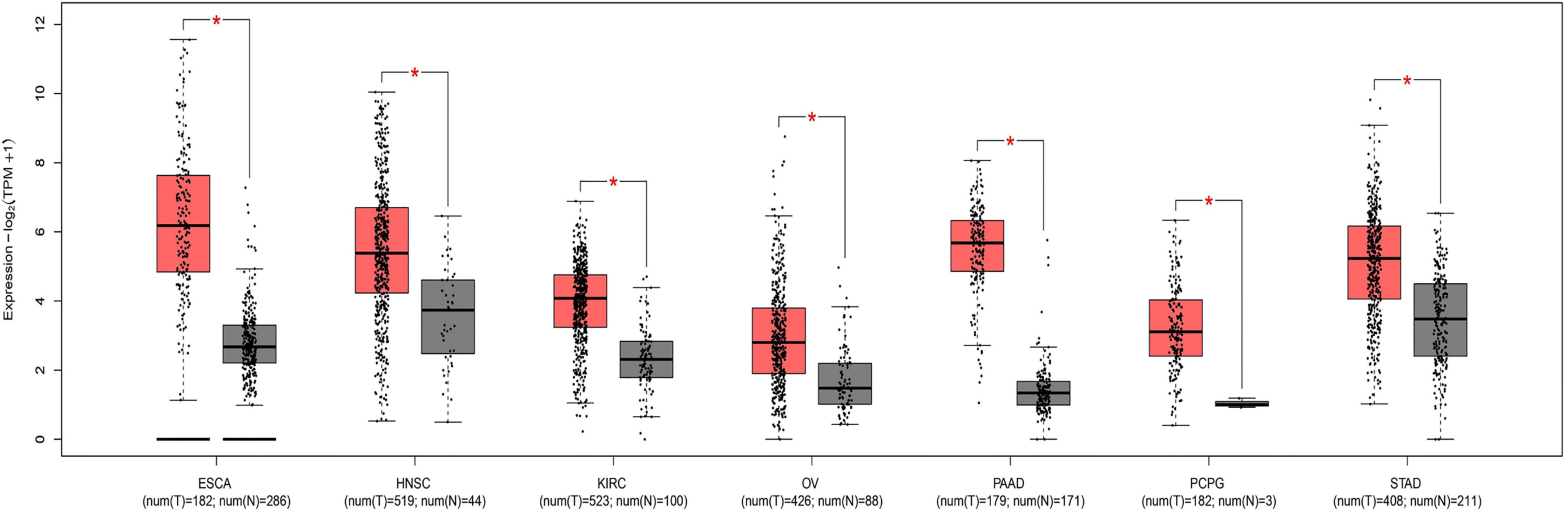
The expression of ANO1 was up-regulated in various malignant tumor ESCA Esophageal carcinoma; HNSC, Head and Neck squamous cell carcinoma; KIRC, Kidney renal clear cell carcinoma; OV, Ovarian serous cystadenocarcinoma; PAAD, Panecreatic adenocarcinoma; PCPG, Pheochromocytoma and Paraganglioma; STAD, Stomach adenocarcinoma. * indicates that the expression of ANO1 is statistically significant between groups.

**Figure 3:**
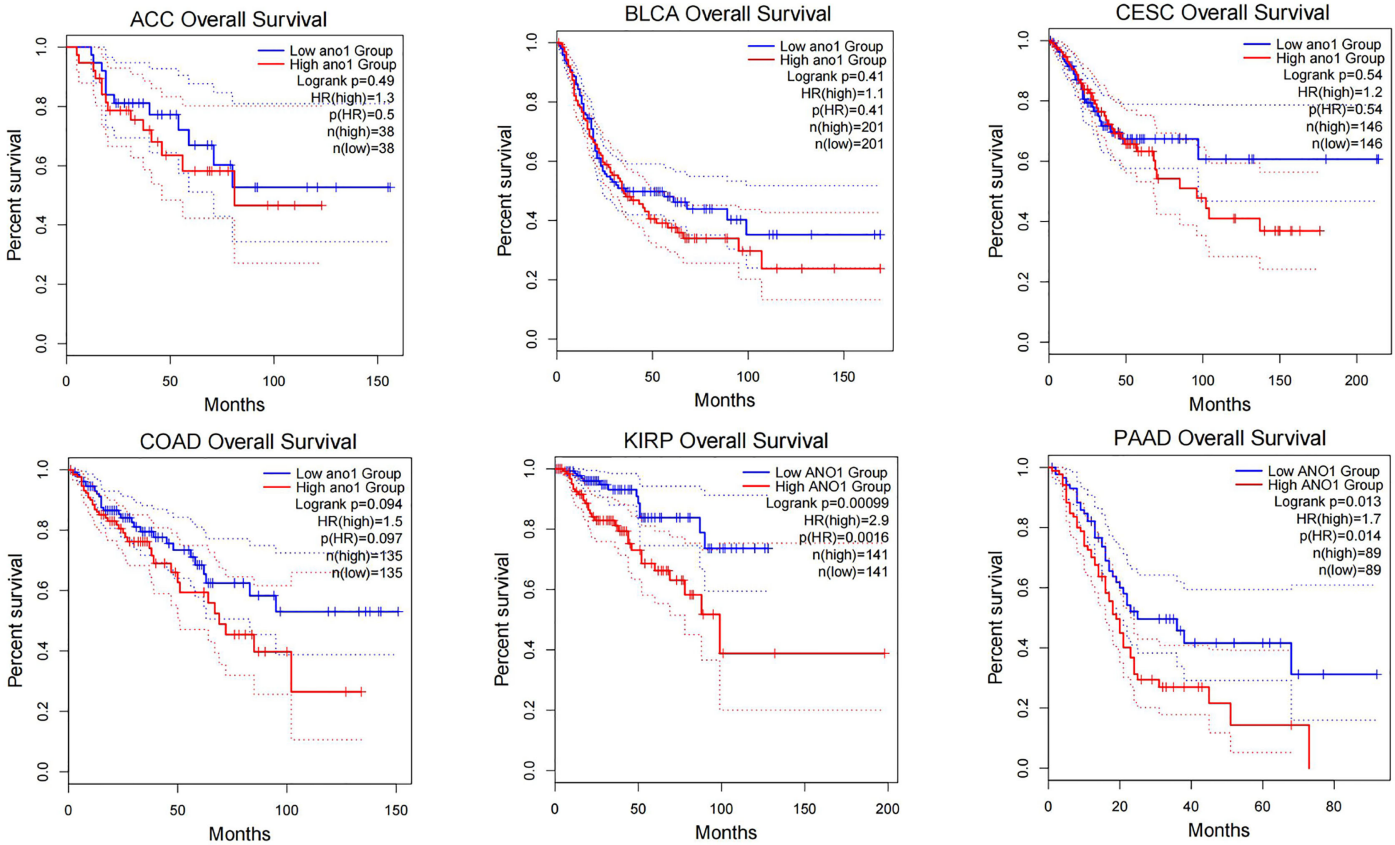
The increased expression of ANO1 is associated with poor prognosis in many malignat tumors ACC, Adrenocortical carcinoma; BLCA, Bladder Urothelial Carcinoma; CESC, Cervical squamous cell carcinoma and endocervical adenocarcinoma; COAD, Colon adenocarcinoma; KIRP, Kidney renal papillary cell carcinoma.

Through research on Human Protein Atlas and GEPIA database, ANO1 overexpression in various malignancies tissues (**Figure 1**). At the same time, up-regulation of ANO1 expression is associated with a worse prognosis in many malignant tumors (**Figures 2**, **3**). ANO1 was reported as a promising biomarker of malignant potential, stage progression, and prognosis, aiding the monitoring of certain malignancies.

The tumor growth and metastasis were observed by transplantation of nude mice and tumor cell culture *in vivo* and vitro. **Table 1** summarizes the role of ANO1 in different tumors. ANO1 was significantly upregulated in various malignant tumors and promotes the invasion of cancer cells. ANO1 overexpression is associated with lymph node metastasis, disease grading and poor prognosis in gastric cancer, liver cancer, and colon cancer (28, 36, 40). Overexpression of ANO1 promoted the growth and invasion of lung cancer, Head and Neck squamous cell carcinoma (HNSCC), breast cancer and pancreatic cancer through the epidermal growth factor receptor(EGFR)signal pathway (11, 22, 39). Especially in breast cancer cells, ANO1 and EGFR-signal transducer and activator of transcription 3(STAT3)signaling pathways form a positive feedback path to promote the proliferation and growth of breast cancer cells. At the same time, ANO1 also activates the Ras-Raf-MEK-ERK1/2 signaling pathway to promote HNSCC and colon cancer (22, 28). ANO1 can regulate the tumor growth factor-β(TGF-β) signaling pathway to promote Esophageal squamous cell carcinoma (ESCC) proliferation, migration, and invasion (17). Silencing or inhibition of ANO1 upregulates tumor necrosis factor-α (TNF-α) expression in prostate cancer cells (27). ANO1 can promote Hepatocellular carcinoma (HCC) and Ovarian cancer metastasis by activating the phosphatidylinositol three kinase-protein kinase B (PI3K-AKT) signaling pathway (23, 41). Meanwhile, ANO1 participates in immune escape in gastrointestinal stromal tumors (42). Finally, neutralization of ANO1 activity with gene knockdown or ANO1 inhibitors has shown anti-neoplastic actions both *in vivo* and *in vitro* models of cancer (**Table 1**).

**Table 1:** The role of ANO1 in different malignant tumors.

Cancer type	Author/year	Tumor cells	ANO1 Overexpression				ANO1 Inhibition				Clinical outcome of ANO1 over expression
			P	M	I	A	P	M	I	A	
**HNSCC**	Ayoub (7) (2010)	HEP-2, SCC-25		**+**	**+**	**+**		**-**			It is related to DNA methylation, 11q13 chromosome amplification, EGFR signaling, MAPK signaling; Its overexpression association with poor prognosis
	Duvvuri (8) (2012)	UM-SCC1	**+**				**-**			
	Ruiz (9) (2012)	BHY		**+**			**-**		**-**	
	Dixit (10)(2015)	PE/CE-PJ34	**+**				**-**			
	Bill (11) (2015)	Te11	**+**							
	Reddy (12) (2016)	SCC-25, CAL-27 CAL-33, BHY		**+**						
	Wanitchakool (13) (2017)	BHY, CAL-133, HT-29, T84	**+**	**+**						
	Finegersh (14) (2017)	NOK	**+**							
	Hermida (15) (2018)	HPV(-)patient tissue	**+**	**+**	**+**					
**ESCC**	Shi (16) (2013)	KYSE30, KYSE510	**+**								It is associated with 11q13 chromosome amplification and TGF-βsignaling; correlation with lymph node metastasis and stage;
	Yu (17) (2019)	kyse30, kyse70, KYSE140, KYSE180	**+**	**+**	**+**		**-**			
**Breast cancer**	Britschgi (18) (2013)	ZR75-1, HCC1954, MDA-MB-415	**+**				**-**	**-**		**+**	It is associated with 11q13 chromosome amplification, EGFR signaling, PI3K-AKT-mTOR signaling, EGFR-STAT3 signaling; and association with poor prognosis;
	Wu (19)(2015)	Patient tissue	**+**				**-**			**+**
	Fujimoto (20) (2018)	YMB-1, BT-549 MD, A-MB-453								
	Wu (21) (2017)	MCF7, MDA-MB-453	**+**							
	Wang (22) (2019)	Mcf7, T47d	**+**							
**Ovarian cancer**	Liu (23)(2018)	SKOU3, ES2	**+**		**+**		**-**		**-**		It is associated with PI3K-AKT signaling and related to the clinical FIGO stage and grade;
**Prostate cancer**	Liu (24) (2014)	PC3, LNCaP, DU145	**+**	**+**	**+**		**-**	**-**	**-**		Its correlation with PI3K-AKT signaling, TGF-a signaling, and related to tumor stage;
	Cha (25) (2015)	PC3, LnCaP, RWPE-1,	**+**	**+**			**-**	**-**		
	Seo (26) (2015)	PC3, HT29, DU145					**-**	**-**	**-**	**-**
	Song (27) (2018)	PC3					**-**	**-**		**+**
**Colorectal cancer**	Sui (28) (2014)	SW620, HCT116, LS174t	**+**	**+**			**-**	**-**	**-**	**+**	It is associated with 11q13 chromosome amplification, MAPK signaling, Wnt/β-catenin signaling, AKT-ERK signaling, EGFR-MAPK signaling; and correlation with lymph node metastasis and stage;
	Mokutani (29)(2016)	DLD1, HCT116					**-**		**-**	
	Jiang (30) (2019)	Caco-2, SW620, HCT116, SW480, LoVo					**-**	**-**		
	Park (31) (2019)	HTC116					**-**	**-**	**-**	**+**
**Lung cancer**	Jia (32)(2015)	NCIH520, H1299, GLC82	**+**		**+**		**-**		**-**		It is associated with EGFR-MAPK signaling
	Hu (33) (2019)	H1299	**+**	**+**	**+**		**-**	**-**	**-**	
**Glioma**	Liu (34) (2014)	U87MG	**+**	**+**	**+**						It is associated with NF-κB signaling, MAPK signaling, and ANO1 channel activities
	Lee (35) (2016)	U251, T98g, U138	**+**	**+**				**-**	**-**	
**Gastric cancer**	liu (36) (2015)	AGS, BGC823	**+**	**+**	**+**						Its correlation with TGF-β signaling and related to lymph node metastasis, stage and poor prognosis;
	Cao (37) (2017)	AGS, MKN45, MKN28, BGC823	**+**	**+**	**+**			**-**	**-**	
**Pancreatic** **adenocarcinoma**	Sauter (38) (2015)	BXPC3, ASPC1		**+**							Its correlation with EGFR signaling and association with lymph node metastasis, stage;
	David (39) (2019)	ASPC1		**+**	**+**					
**Hepatocellular** **carcinoma**	Deng (40)(2016)	SMC7721	**+**	**+**							Its correlation with PI3K-AKT, MAPK signaling, and related with lymph node metastasis and stage; and association with poor prognosis;
	Zhang (41) (2020)	Patient tissue	**+**	**+**	**+**	**-**	**-**	**-**	**-**	**+**

P, proliferation; M metastasis; I, invasion; A, apoptosis; + promotes; - inhibit.

ANO1, a calcium-activated chloride channel; AKT, Protein Kinase B; EGFR, Epidermal Growth Factor Receptor; ERK, Extracellular Regulated Protein Kinases; ESCC, Esophageal squamous cell carcinoma; HPV, Human Papilloma Virus; MAPK, Mitogen-Activated Kinase; MEK, Mitogen-Activated Extracellular Signal-Regulated Kinase; MTOR, Mechanistic Target of Rapamycin; NF-κ B, Nuclear Factor Kappa-B; PI3K Phosphatidylinositol 3 Kinase; STAT3, Signal Transducer and Activator of Transcription three; TGF-a, Tumor Necrosis Factor-α; TGF-β, Tumor Growth Factor-β; FIGO, Federation International of Gynecology and Obstetrics.

In summary, it shows that ANO1 is related to cancer risk and progression in the following aspects: 1) its upregulation in a variety of tumor tissues; 2) its correlation with tumor proliferation, invasion depth, lymph node metastasis, distant metastasis, and apoptosis;3) the gene expression level of ANO1 is closely related to tumor size and differentiation; 4) its association with advanced stage and poor prognosis; 5) its interacts with various oncogenic signaling networks; 6) it regulate tumor microenvironmental tumorigenic signaling pathways.

The expression of ANO1 seems to be controlled by various molecules and stimuli. Different cells have different regulatory mechanisms and signaling pathways. ANO1 can participate in various signal pathways not only with the interaction with various proteins but also with the ion pathway that regulates the ion homeostasis of tumor cells, thereby promoting the critical functions of tumor cells. Further studies to clarify the molecular mechanism are required, for up-regulated tumors, promoting proliferation, metastasis, invasion, and avoiding apoptosis. The mechanism of ANO1’s involvement in malignant tumors will be reviewed.

## The Mechanism of Ano1 Involved in Cancer

**Figure 4:**
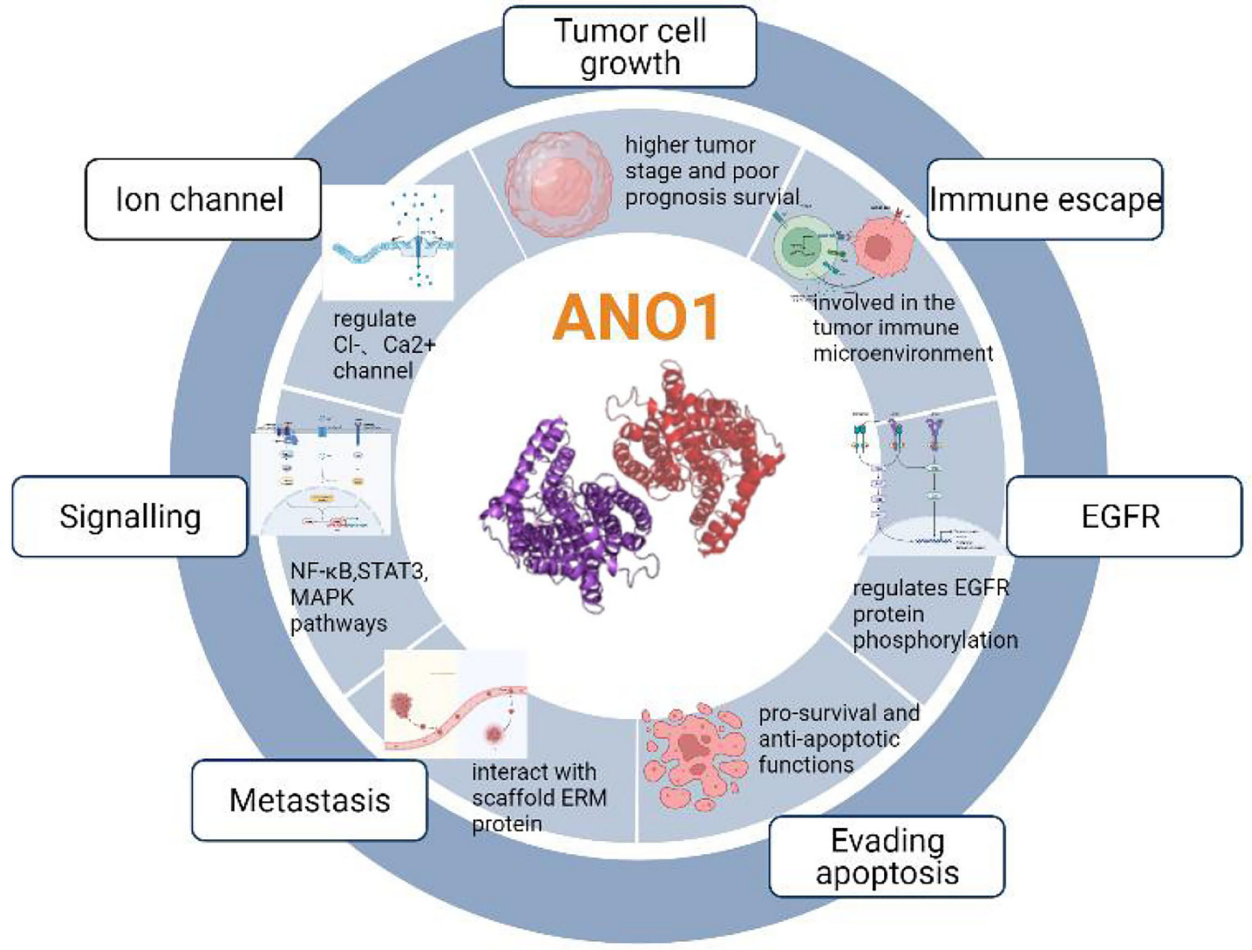
The involvement of ANO1 in tumorigenesis.

Previous studies have shown that ten biological functions formed during the multi-stage development of human tumors constitute the characteristics of cancer (43). These ten characteristics include self-sufficiency in growth signals, insensitivity to anti-growth signals, evading apoptosis, Limitless replicative potential, sustained angiogenesis, tissue invasion & metastasis, genome instability and mutation, tumor-Promoting inflammation, deregulating cellular energetics, avoiding immune destruction. The involvement of ANO1 in tumorigenesis is currently related to the following aspects (**Figure 4**).

### ANO1 Overexpression Is Involved in the Unlimited Replication and Proliferation of Tumor Cells

ANO1 is upregulated in various tumor tissues and correlates with proliferation, invasion depth, lymph node metastasis, distant metastasis, and apoptosis. Multiple mechanisms can explain the overexpression of ANO1. ANO1 is located on human chromosome 11q13, commonly amplified in several cancers, and participates in tumorigenesis, invasion, and migration. Therefore, the most common mechanism of ANO1 overexpression in cancer amplifies the 11q13 locus (10, 44). This expansion has been described in some cancer types, including breast, gastric, esophageal, and lung carcinoma. However, it is essential to mention that 11q13 amplification resulted in a higher TMEM16A expression in human breast cancer and HNSCC than in non-11q13-amplified tumors. Meanwhile, ANO1 is sufficient to promote cell proliferation without 11q13 amplification, which shows that 11q13 gene amplification is not the only factor that promotes ANO1 overexpression (18).

### ANO1 Interacts With Various Proteins to Promote Tumor Proliferation and Invasion

**Figure 5:**
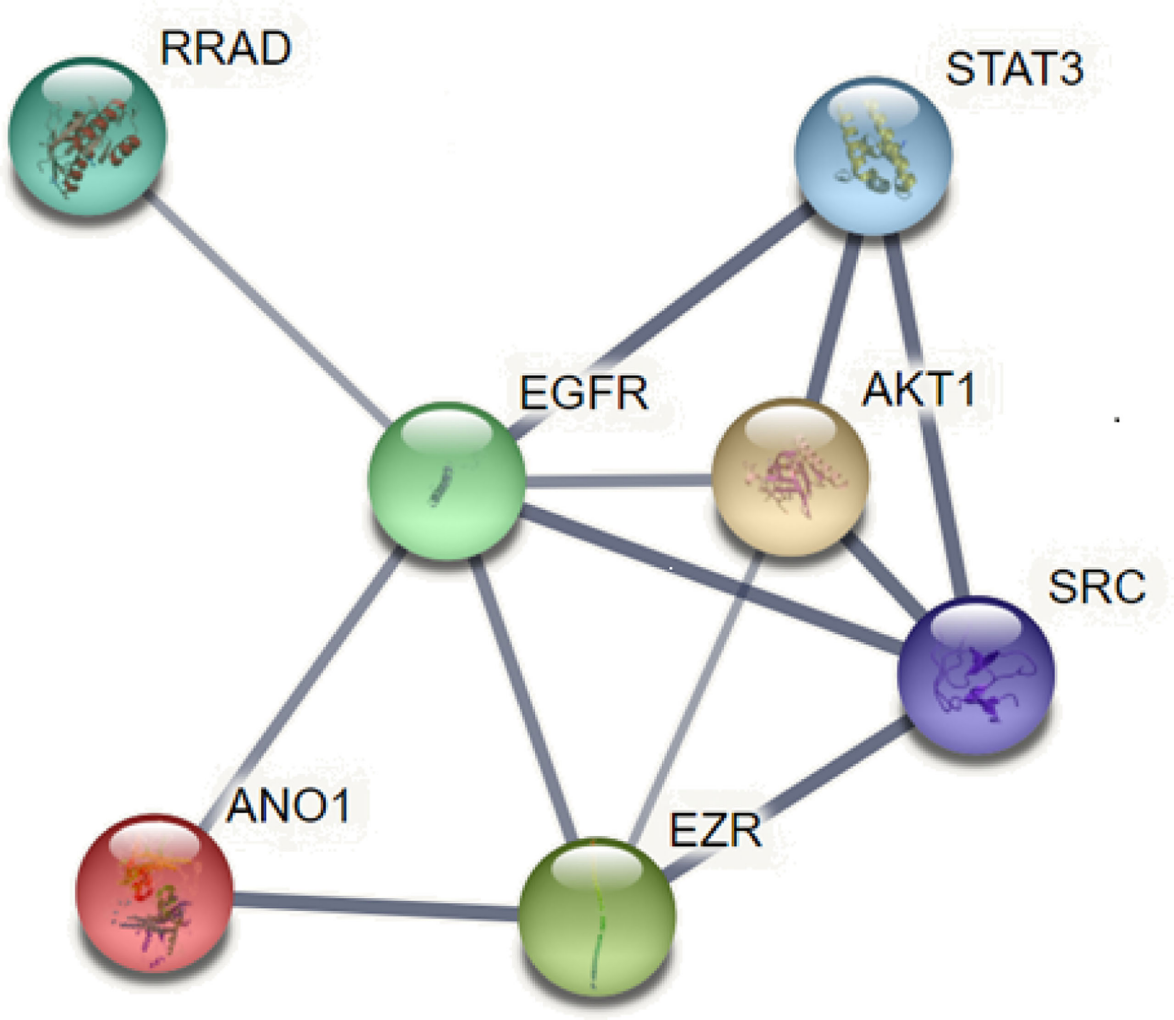
ANO1 interacts with various proteins.

EGFR is overexpressed in many tumors such as HNSCC, breast cancer, and pancreatic cancer and is involved in tumorigenesis. ANO1 was also observed as a core protein in the STRING protein network pathway, which can regulate EGFR constitutive protein phosphorylation and related signal pathways, such as protein tyrosine kinase (SRC), protein kinase B (AKT), promote the proliferation of cancer cells (18, 45) (**Figure 5**). The interaction between ANO1 and EGFR can also increase ANO1 protein stability. At the same time, ANO1 also has a significant effect on the remodeling of EGF-induced protein phosphorylation. The study also found that the AKT and extracellular regulated protein kinases (ERK) signaling pathways are not affected by the expression of ANO1, which was further confirmed by the STRING pathway of ANO1. So, the relationship between the expression of ANO1 and EGFR in different signaling pathways is different (39). Therefore, it is crucial to understand how ANO1 regulates the EGFR signaling pathway.

Recent data in pancreatic cancer show that ANO1 is necessary to promote EGF-induced EGFR signal transduction, EGF-mediated Ca2^+^ storage depends on ANO1, and the ANO1-mediated Ca2^+^ signaling pathway regulates EGF-induced may be involved in the proliferation of pancreatic cancer cells, and may dramatically impact the migration (46, 47). It can be seen that ANO1 provides a new entry point for treating pancreatic cancer.

In the study of breast cancer cells, EGF initiates EGFR/STAT3 signal transduction, which promotes the overexpression of ANO1, while ANO1 overexpression further activates the EGFR-STAT3 signal transduction of breast cancer cells. The positive feedback loop between ANO1 and EGFR-STAT3 promotes the proliferation and migration of breast cancer cells. Knockout of ANO1 will reduce the phosphorylation of EGFR in breast cancer cells, further inhibit the activation of AKT, SRC, and ERK, and reduce the autocrine of EGFR ligands, EGF and TGF-β, suggesting that ANO1 may increase EGFR ligands The autocrine activates EGFR signal transduction (22). Therefore, we considered that ANO1 could activate the EGFR signaling pathway by increasing EGFR expression, phosphorylation, and EGFR ligand autocrine. More research is needed to confirm the connection between ANO1 and EGFR.

ANO1 also regulates cell morphology and volume and directly interacts with scaffold ERM protein (Ezrin/Radixin/Moesin) (48) (**Figure 5**). The ERM protein connects the plasma membrane with the actin cytoskeleton, affects the cell morphology of cancer cells, and regulates the movement, metastasis, and invasion of tumor cells. At the same time, ERM can be activated by many ligands as scaffold proteins to promote the signal transduction of membrane proteins, including EGF ligands, to promote the deformation and invasion of cancer cells. Studies have found that ANO1 may interact with EGFR to recruit ERM proteins, promoting EGFR signal transduction and activation of the MAPK/AKT pathway (11). The ANO1 inhibitor Des inhibited the activity and migration of non-small-cell lung carcinoma by decreasing the levels of Ano1, p-ERK1/2 and p-EGFR (49).

### ANO1 Activates Multiple Signaling Pathways Involved in Tumor Proliferation, Migration and Invasion

**Figure 6:**
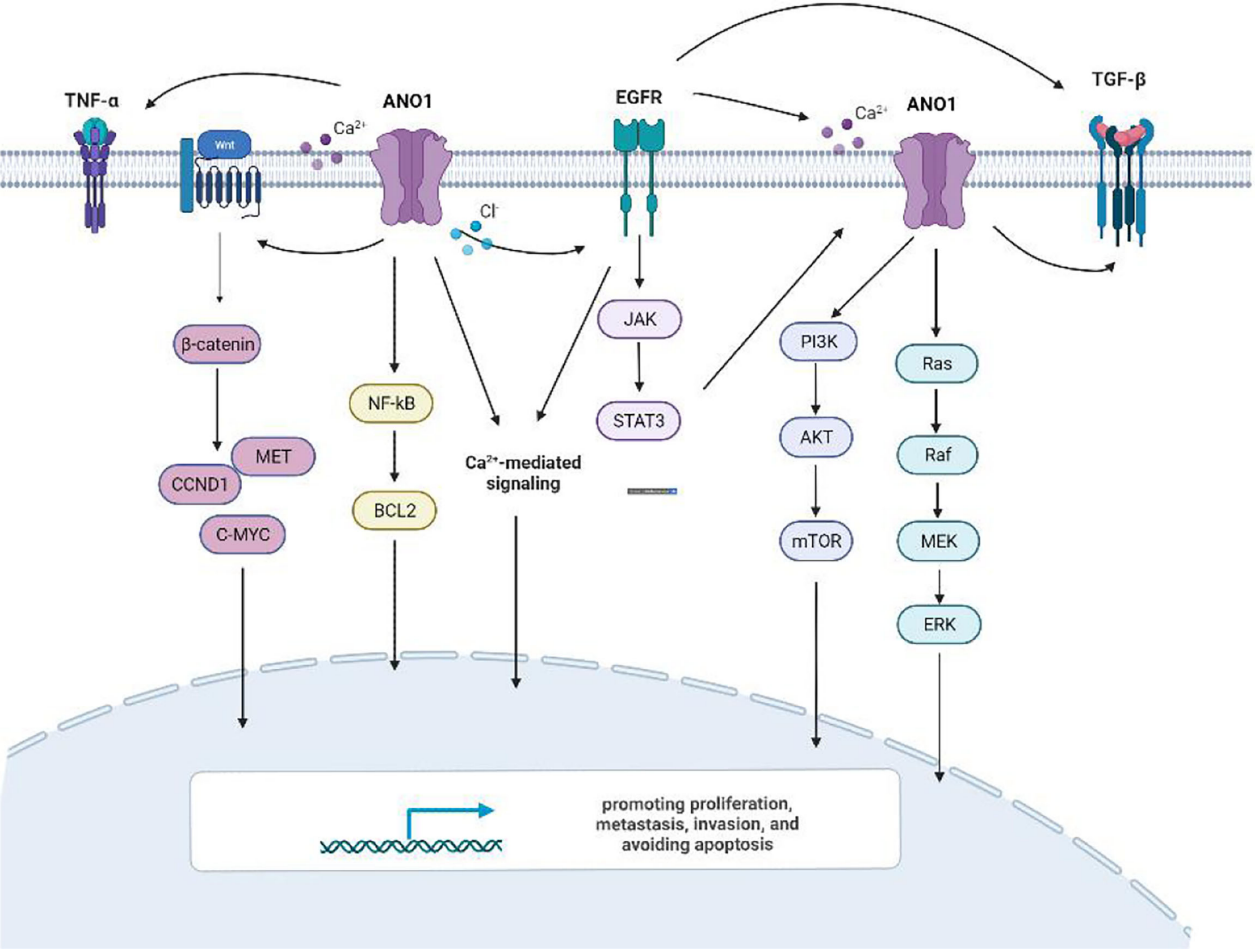
ANO1activating multiple signaling pathways.

There is ample evidence that ANO1 triggers numerous signaling pathways and stimulates many biological effects in various cell types (**Figure 6**). Overexpression of ANO1 promoted the growth and invasion of lung cancer cells through the EGFR-MAPK signal pathway (33). ANO1 also activates the Ras-Raf-MEK-ERK1/2 signaling pathway to promote tumor cell growth of HNSCC (8). At the same time, ANO1 can regulate the TGF-β signaling pathway to promote ESCC cells proliferation, migration, and invasion (17). Silencing or inhibition of ANO1 inhibits cell growth, induces apoptosis, and upregulates TNF-α expression in prostate cancer cells (22). Calcium ions (SOCE) that activate ANO1 and ANO1-dependent storage can enter cells and regulate EGFR ligands to promote pancreatic cancer cell migration (39). The higher the expression level of ANO1 in HCC is accompanied by higher tumor grades, lesions, metastases, and a poor prognosis (40, 41). ANO1 can promote HCC metastasis by activating EGFR phosphorylation and subsequent PI3K-AKT signaling pathway (41). In breast cancer cells, ANO1 and EGFR-STAT3 signaling pathways form a positive feedback path to promote the proliferation of breast cancer cells (22). At the same time, ANO1 mediated the PI3K-AKT-mTOR and JAK-STAT3 signaling pathways of HER2-positive breast cancer cells by regulating intracellular Cl^-^ to participate in HER2 transcription and promote tumor proliferation (21). Inhibit of ANO1 can inhibit PI3K-AKT signaling and inhibit the Ovarian cancer cell proliferation and invasion (23). ANO1 affects GBM cell proliferation by regulating NF-κB mediated genes. In the meantime, it is involved in cell proliferation cyclin D1, cyclin E and c-myc, and matrix metalloproteinase (MMP) 2 and MMP 9 induce GBM cell invasion and migration (34, 35). Higher expression of ANO1 was related to gastric and colorectal cancer lymph node metastasis, advanced tumor stage and poor prognosis. Knockout or pharmacological inhibition of ANO1 inhibits the proliferation and induces apoptosis of colorectal cancer cells *via* the Wnt/β-catenin signaling pathway (50).ANO1 is a direct target and negatively correlates with miR-9, miR-144 and miR-381 (30, 31, 37). Overexpression of miR-9 suppressed the expression of p-AKT, cyclin D1, and p-ERK. MiR-144 can inhibit colorectal cancer by inhibiting the EGFR-ERK signaling pathway targeting ANO1. Meanwhile, miR381 directly targets ANO1 to regulate the TGF-β pathway. ANO1 also has been considered an independent prognostic factor affecting the prognosis of gastric cancer patients.

ANO1 participates in various signaling pathways to promote proliferation, metastasis, and invasion. The signal pathway involved in each tumor is not unique, and one signal pathway can correspond to multiple tumor types. To this end, it would be significant to determine the ANO1 receptor and clarify its signaling pathway in tumor promotion.

Meanwhile, ion channels also participate in ANO1 regulated signaling pathways. Some scholars believe that ANO1 may regulate EGF through Ca2^+^ signaling to promote pancreatic cancer cell migration (39). Some scholars have also found that silencing the expression of ANO1 reduces the intracellular Cl^-^, thereby reducing the secretion of TGF-β and inhibiting the metastasis and invasion of gastric cancer cells (36). At the same time, in breast cancer cells, a decrease in the activity of the Cl^-^ channel can down-regulate the EGFR signaling pathway (18). The mechanism of ANO1 in each tumor is different, so that it may constitute the heterogeneity of ANO1 overexpressing cancer cells. However, further studies are needed to clarify whether specific cancer types have unique ionic states.

### Alteration of ANO1 Protein Level and Ion Channel Activity Promotes Cancer Cell Proliferation and Migration

Many studies are researching how the increase in ANO1 protein level and channel activity is involved in the proliferation and migration of malignant tumor cells. The gating characteristics of ANO1 are complex because it involves the increase in intracellular calcium ion concentration, membrane depolarization, and the interaction between extracellular chloride or transition anions and intracellular protons (11).

ANO1 contains ten putative transmembrane domains, intracellular NH2 and COOH end, and two calmodulin-binding domains (51). E702 and E705 are presumed to be Ca2+ binding sites, and ANO1 expresses multiple splice variants with variable sensitivity to cytoplasmic Ca2^+^. Studies have found an X-ray crystal structure (52) (a fungus with Ca2^+^ activated lipid interfering enzyme activity nhTMEM16) ANO1 subtype has 39-42% homology with mammalian ANO1 (53, 54). nhTMEM16 contains ten transmembrane subunit fragments and six residues (including glutamic acid and aspartic acid). Exploring the fungal model of ANO1 will help determine the Ca2^+^ binding site. However, the exact Ca2+ binding mechanism of ANO1 still needs to be further explored, even identifying the Ca2+ binding site and ionic conductive hole of ANO1.

For the structural study of ANO1, the current research shows two main ion phenomena: A) ANO1 is activated by voltage when Ca2^+^ is absent in tumor cells. B) After reducing the concentration of extracellular Cl^-^, the conductivity of Cl^-^ in ANO1 decreases (55). The intracellular Ca2^+^ signaling is an essential regulator of cell proliferation, and ANO1 can control intracellular Ca2^+^ levels by regulating Ras-Raf-MEK-ERK1/2, PIK3-AKT, and DAG-IP3 receptor signals (11, 51). In many systems, the transient increase of intracellular Ca2^+^ and the continuous activation of the Ras-Raf-MEK-ERK1/2 pathway are the central links in cell proliferation (51, 56, 57). The steady-state regulation of Ca2^+^ and chloride ion conductance in ANO1 channel activity is also involved in the EGF-induced EGFR pathway. In addition, Cepharanthine significantly inhibits cell proliferation, migration and induces apoptosis in lung adenocarcinoma cells *via* endogenous ANO1 currents (58). Change and regulation of Ca2^+^ concentration play an essential role in the ANO1 function of cancer cells.

At the same time, the Cl^-^ channel is the key to maintaining cell volume, so it is crucial in tumor cell migration and invasion (44, 59, 60). In cancer, related changes in intracellular water content can regulate cell volume, and the resulting morphological changes are critical to cell division, migration, invasion, and prevention of cell death (61, 62). The osmotic adjustment represents a mechanism that links ion flux to different cancer cell functions. Some reports have observed that inhibiting ANO1 can affect the size or morphology of cancer cells (7, 60, 63). Furthermore, the activation of Cl- concentration in ANO1 can regulate cell proliferation, and the overexpression of cell ANO1 protein and the increase of channel activity may lead to changes in Cl- concentration. Intracellular Cl^-^ can function as a second messenger, regulating various proteins in many signaling pathways (48, 64). So far, it is still unclear whether the overexpression of ANO1 will increase or decrease the concentration of Cl^-^. Some scholars have put forward the hypothesis: If ANO1 overexpression can reduce the concentration of Cl^-^, it may be by increasing the outflow of Cl- in the cell. Experiments have shown that the Cl^-^ channel associated with glioblastoma can be transported to the plasma membrane and act as an ion channel, explicitly participating in tumorigenesis (65).

Next, we determined that the homeostasis regulation of Ca2^+^ and Cl^-^ currents also has a more significant effect on ANO1 in cancer cells. Some scholars established a 12-state Markov chain model by studying the mechanism of membrane potential, Ca^2+^, and Cl^-^ on ANO1 ion channel gating (54). This model interprets the activation of ANO1 as 2 Ca^2+^ dependent on the membrane voltage, directly and continuously coupled with the external Cl^-^ dependent on the membrane potential, and transition into a voltage-dependent state. This model assumes that further experiments prove no significant change in extracellular Cl^-^ affinity for ANO1 Ca2^+^. However, experiments have also found that extracellular Cl^-^ can promote voltage-dependent activation by stabilizing the open configuration induced by Ca^2+^. The establishment of the model helped to understand the ion channel mechanism of ANO1. However, the related mechanism of ANO1 channel activity and tumor also needs to be confirmed in other models.

While most reports have confirmed the role of ANO1 overexpression in cancer cell proliferation and migration, there is insufficient information on the role of ANO1 in cell proliferation and migration, mainly due to the increase in ANO1 protein level or the increase in channel activity. Studies have found that reducing the expression level of ANO1 protein may be more critical than blocking the activity of ANO1 channels (66).

### ANO1 Induces Tumor Cells to Escape Apoptosis and Regulate the Cell Cycle

Apoptosis is a highly regulated process of cells, essential for cell growth and tissue development (67, 68). Exogenous pathways can trigger apoptosis, including extracellular, pro-apoptotic, ligand-receptor interaction, and endogenous (69). In breast cells, the expression of ANO1 increased anti-apoptotic proteins BCL2 and MCL-1, indicating that ANO1 has pro-survival and anti-apoptotic functions (18). TNF-α expression was negatively correlated with ANO1 expression in prostate cancer cells. Inhibition of ANO1 can activate the TNF-α signaling and induce apoptosis *via* caspase activation (27). ANO1 also may regulate HCC cell apoptosis through the control AKT/MAPK signaling pathway (41). ANO1 expression correlates with increased ERK 1/2 activity and less apoptotic activity in HNSCC (70). ANO1 inhibitors can cause the overexpression of miR9 to inhibit the proliferation of colorectal cancer cells and induce cell cycle arrest at the GO/G1 phase leading to cell apoptosis (31). At the same time, inhibiting ANO1 can reduce the chloride channel activity and the expression of EGFR and calmodulin dependent kinase (CAMKII), thus promoting breast cancer cell apoptosis (18). Therefore, Suppression of ANO1 overexpression induces apoptosis and offers a promising new modality for the future treatment of malignant tumors.

The G1 phase arrest of the cell cycle can slow cell proliferation. Studies have found that inhibiting ANO1 can induce cell cycle G2/M phase arrest. Nevertheless, it does not affect cell cycle distribution (71). Meanwhile, changes in ANO1 expression affect proteins used explicitly for transcription or cell division. Such as cyclin A2, cyclin D1, and cyclin E. Up-regulation of cyclin-dependent kinases 1 and 2 (CDK1 and CDK2) in cancer cells can cause an increase in the expression of ANO1 (34, 52). The above data indicate that ANO1 may be involved in the cell cycle process, but it remains to be clarified how ANO1 induces tumor cell cycle arrest. Existing studies have found that the ANO1 inhibitor Caccinh-A01 may reduce the chloride current in gastric cancer cells and cause the G1 phase of the cell cycle block (72). At the same time, research on colorectal cancer found that cyclin D1 protein expression was positively correlated with ERK1/2 and ANO1.ANO1 inhibits G1 to S phase progression by down-regulating the expression of the ANO1, which is consistent with the decreased expression of cyclin D1 (28). Suppressing ANO1 expression also had significantly lower cyclin D1, leading to changes in p27^Kip1^ distribution and correlated with a cell cycle arrest phenotype in HNSCC (8, 73).

### ANO1 Is Involved in Tumor Immune Evasion

Tumor immune escape signifies that tumor cells escape recognition and attack by the immune system through various mechanisms to survive and proliferate (74). Future development of cancer immunotherapy depends on the crosstalk between cytokines produced by the tumor microenvironment and biology. In the study of gastrointestinal stromal tumors, ANO1 gene expression was significantly negatively correlated with plasma cells and activated memory CD4^+^ T cells, implying that ANO1 may be involved in the functional inhibition of helper T cells (42). We consider that ANO1 may be related to the immune microenvironment of malignant tumors. These specific mechanisms also remain to be systematically investigated.

## Perspectives and Future Directions of Ano1

As a calcium-activated chloride channel, ANO1’s expression is affected by various molecular mechanisms. Current research shows that ANO1 may be involved in malignant tumours’ occurrence, development, and metastasis. Finally, novel, more effective, and safer ANO1-centered therapeutic interventions may open new horizons in oncotherapy and provide a new cancer treatment strategy. Some compounds such as cepharanthine (58), Ani9 (36, 75), cinobufagin (76), luteolin (77), Aa3 (78), theaflavin (79), 2-aminothiophene-3-carboxamidederivatives (80), matrine (76), have been identified as inhibitors of ANO1 Agent. It has been validated in multiple *in vitro* and vivo tumor models (**Table 2**).

**Table 2:** ANO1 inhibitor and its antitumor mechanism.

ANO1 inhibitors	Tumor cells	Mechanisms	Year
Cepharanthine (58)	LA795	reduced ANO1 channel activity	2021
Diethylstilbestrol (DES) (49)	PC9	1. reduced both ANO1 channel activity and cell viability 2. reduction of p-ERK1/2 and p-EGFR levels. 3. induced apoptosis by increasing caspase-3 activity and PARP-1 cleavage	2021
Ani9 (75, 80)	PC3, MCF7, BxPC3, DU145, LNCaP, 22RV1	1. reduced the protein levels 2. TNF-α signaling, activation of JNK and JUN	2016 2018
new2-aminothiophene-3-carboxamide derivatives (9c and 10q) (81)	U251	1. suppress ANO1 channel activities 2.combination of ANO1 inhibitor (9c or 3) and temozolomide (TMZ) brings about remarkable synergistic effects	2020
			
T16A(inh)-A01 (38, 82)	Panc-1, Mia PaCa2, Capan-1, AsPC-1, BxPC-3, HEK-293T, UM-SCC1, T24 DU145, LNCaP, 22RV1	1. suppress ANO1 channel activities 2. regulated ERK1/2 activation and cyclin D1 3. TNF-α signaling, activation of JNK and JUN	2012 2018
Arctigenin (83)	LA795	Inhibited MAPK pathway	2020
CaCCinh-A01 (38)	Panc-1, Mia PaCa2, Capan-1, AsPC-1, BxPC-3, DU145, LNCaP, 22RV1	TNF-α signaling, activation of JNK and JUN	2015 2018
NS3728 (38)	Panc-1, Mia PaCa2, Capan-1, AsPC-1, BxPC-3	altered ATP-induced [Ca2+]i signals	2015
Cinobufagin (76)	CAL-27	1. reduced phosphorylation of STAT3 2. induced caspase-3 activation and PARP cleavage	2021
Luteolin (77)	PC3	inhibited ANO1 channel activity and protein expression levels	2017
Matrine (84)	LA795	NR	2019
benzophenanthridine alkaloids (85)	LA795	inhibited ANO1 channel activity	2020
Avermectins (86)	LA795	NR	2020
Aa3 (78)	A549, NCI-H460	NR	2020
Theaflavin (79)	LA795	block the ion conduction pore	2021

NR, not reported.

Simultaneously, microRNAs (miRNAs) can also directly target and inhibit ANO1 expression suppressing tumor cells. MiRNA is small non-coding RNAs with about 22 nucleotides. miRNAs play an essential regulatory role in various cancers, including gastric cancer, pancreatic cancer, and hepatocellular carcinoma. They bind to the 3’-untranslated region (3’-UTR) of target genes, resulting in irreversible inhibition of transcription or translation. Because abnormally expressed miRNA also contributes to cancer cells’ proliferation, apoptosis, and metastasis (87–89). Moreover, miRNA expression is considered a promising biomarker of cancer diagnosis, prognosis, and therapy (90).

**Figure 7:**
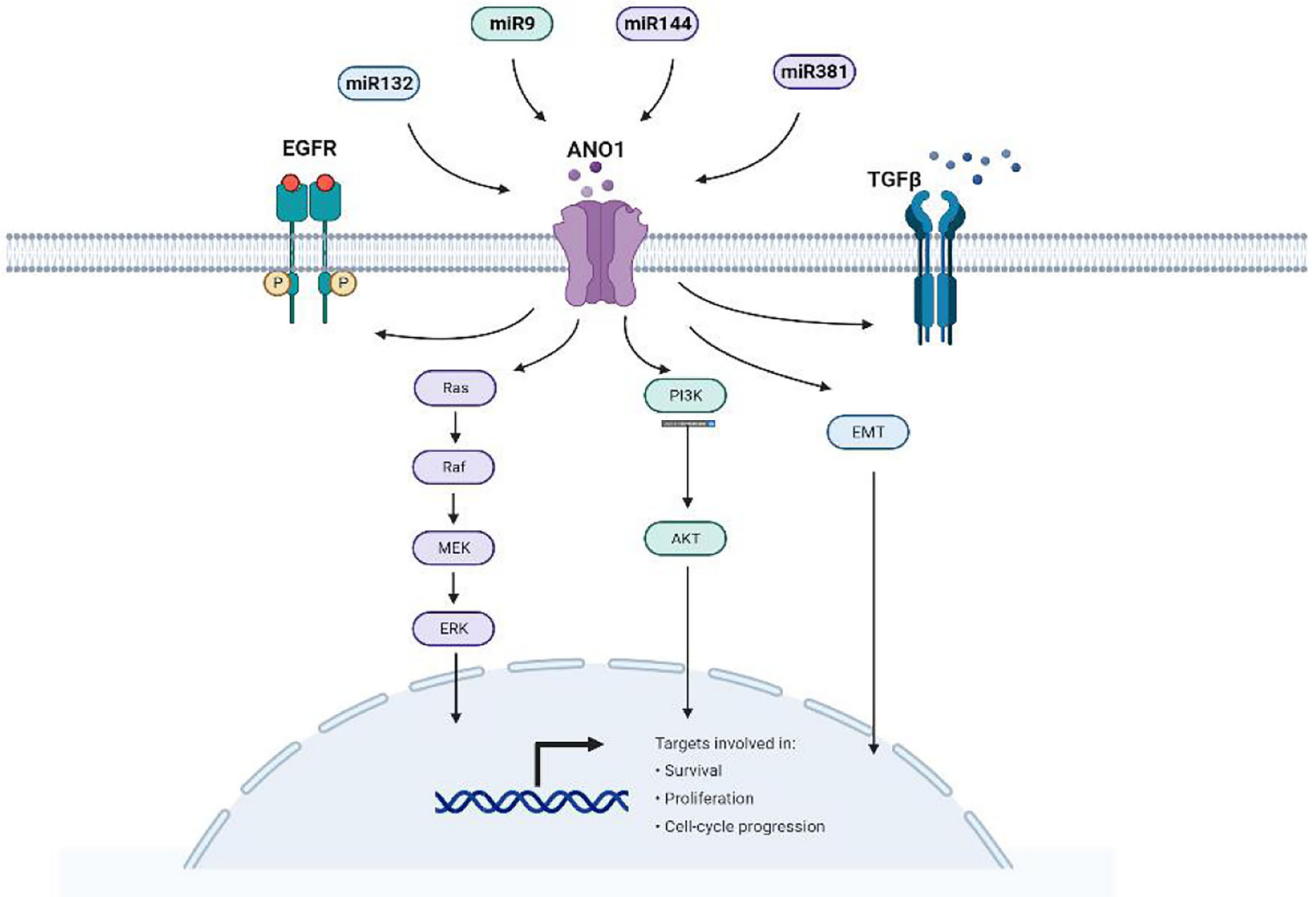
miRNA directly targets ANO1and participates in tumorigenesis.

Existing studies have found that luciferase detection shows that ANO1 is a direct target and is negatively correlated with miR132, miR144, miR381, and miR9 (**Figure 7**). High miR144 and miR9 directly target ANO1 to inhibit colorectal cell proliferation and migration and promote apoptosis (30, 31). miR9 overexpression inhibits ANO1 miRNA and protein expression and inhibits the expression of P-AKT, CD1, and P-ERK proteins in colorectal cancer cells (31). miR381 expression is related to gastric cancer proliferation, lymph node metastasis, advanced stage, and poor prognosis by directly targeting ANO1 to regulate the TGF-β pathway (37). MiR144 also down-regulates the expression of PTEN to activate the Ras-Raf-MEK-ERK1/2 pathway to inhibit breast cell proliferation and survival (30). Exploring the relationship between ANO1 and microRNA has broad prospects for the occurrence and development of tumors.

## Summary

ANO1 has recently attracted considerable attention because of its potential role in cancer chemoprevention and chemotherapy. ANO1 can participate in various signal pathways by interacting with a variety of proteins or by regulating the ion conduction of malignant tumor cell ion homeostasis, thereby affecting the biological behavior of malignant tumors. Moreover, ANO1 expression can constitute a vital diagnosis and therapy monitoring marker. Advances in ANO1 biology and ANO1 inhibitors may hold promise for ANO1 use as a potential cancer biomarker and therapeutic target. The development of ANO1 inhibitors is currently desirable and validated in multiple *in vitro* and vivo tumor models and used in clinical trials. Simultaneously, many questions remain to be answered, yet other fields need additional exploration.

## Data Availability Statement

The original contributions presented in the study are included in the article/supplementary material. Further inquiries can be directed to the corresponding author.

## Author Contributions

All authors made a significant contribution to the work reported, whether that is in the conception, study design, execution, acquisition of data, analysis, and interpretation, or in all these areas; took part in drafting, revising or critically reviewing the article; gave final approval of the version to be published; have agreed on the journal to which the article has been submitted; and agree to be accountable for all aspects of the work.

## Funding

This study was partially supported by China’s National Natural Science Foundation(81900198), the Dalian Science and Technology Innovation Program, and the Natural Science Foundation of Liaoning Province(2020-MS-257).

## Conflict of Interest

The authors declare that the research was conducted in the absence of any commercial or financial relationships that could be construed as a potential conflict of interest.

## Publisher’s Note

All claims expressed in this article are solely those of the authors and do not necessarily represent those of their affiliated organizations, or those of the publisher, the editors and the reviewers. Any product that may be evaluated in this article, or claim that may be made by its manufacturer, is not guaranteed or endorsed by the publisher.
